# Plasmacytic Pleural Effusion as a Major Presentation of Angioimmunoblastic T-Cell Lymphoma: A Case Report

**DOI:** 10.3390/curroncol29100603

**Published:** 2022-10-13

**Authors:** Borui Li, Lin Nong, Jianhua Zhang, Wensheng Wang, Qian Wang, Yang Zhang, Shaomin Ren, Mangju Wang

**Affiliations:** 1 Department of Hematology, Peking University First Hospital, Beijing 100034, China; 2 Department of Pathology, Peking University First Hospital, Beijing 100034, China; 3 Department of Nuclear Medicine, Peking University First Hospital, Beijing 100034, China

**Keywords:** angioimmunoblastic T-cell lymphoma, plasmacytic pleural effusion, reactive plasmacytosis, flow cytometry

## Abstract

Angioimmunoblastic T-cell lymphoma is one of the peripheral T-cell lymphomas. Reactive plasma cells can occasionally be observed in AITL patients’ peripheral blood and bone marrow. Plasmacytic pleural effusion as the presentation of AITL has not been reported before. The mechanisms of plasmacytic pleural effusion are not fully understood. Here we present an 82-year-old male with exuberant plasma cells in his pleural effusion in addition to his peripheral blood and bone marrow aspiration. By presenting this case, we would like to expand the spectrum of disease presentations in AITL and discuss the significance of flow cytometry in the differential diagnosis of pleural effusion. To our knowledge, this is the first case report in the literature, which will be crucial to assist the hematopathologist in accurate diagnosis and treatment.

## 1. Introduction

Angioimmunoblastic T-cell lymphoma (AITL), once considered a benign T-cell proliferation [1], is one aggressive neoplasm. Based on the 2016 revisions of the World Health Organization (WHO) Classification of Lymphoid Neoplasms, the AITL was reclassified as a distinct peripheral nodal T-cell lymphoma [2]. Although it is the second most prevalent PTCL [3], the incidence was only about 0.05 cases per 100,000 person-years [4]. It mainly occurs in elderly patients. Its symptoms and signs include generalized lymphadenopathy, hepatomegaly, splenomegaly, systemic B symptoms of fevers, night sweats and weight loss, rash, and polyarthritis [5].

The architectures of the involved lymph nodes are entirely or partially effaced by a predominantly paracortical infiltrate of neoplastic and inflammatory cells. The peripheral sinus is frequently preserved and dilated. The inflammatory background is composed of a variety of immune cells including plasma cells, B cells, immunoblasts, eosinophils and epithelial cells. The other significant features, including a proliferation of small high endothelial venules (HEVs) and a coarse irregular extrafollicular meshwork of follicular dendritic cells (FDCs), are vital clues in the assistance of diagnosis [6].

In peripheral blood, patients with AITL can present with anemia, lymphopenia and thrombocytopenia. The marked reactive plasmacytosis in peripheral blood and/or bone marrow has been reported in several cases [7,8]. Pleural effusions are not rare conditions in lymphoma patients. However, pleural effusion rich in plasma cells is not common in patients with plasma cell dyscrasia or infectious diseases such as tuberculosis [9,10]. To our knowledge, marked reactive plasmacytosis in pleural effusion had never been observed before in patients with AITL lymphoma. Here, by presenting this case, we would like to introduce a new phenomenon seen in AITL and review the importance of flow cytometry in the differential diagnosis of malignancies.

## 2. Case Presentation

An 82-year-old man presented with fatigue, night sweats, weight loss and lymphadenopathy for three months, without fever or pruritus. CBC showed mild anemia (HGB 113 g/L) and thrombocytopenia (PLT 90 × 10^9^/L). The reactive plasma cell was 5% in peripheral blood. His serum total protein and albumin were within the normal range. Ultrasounds showed multiple lymph node enlargements (cervical, supraclavicular, axillary and inguinal), hepatomegaly and splenomegaly. His PAIgG was positive (78.3%). The bone marrow smear showed marked hyperplasia. Plasma cells accounted for 5%. The immunohistochemistry of the lymph node biopsy was positive for CD20, PAX5 and CD3. Ki65 accounted for 50%. EBV-DNA was positive both in his serum (8.03 × 10^5^ copies/mL) and peripheral lymphocytes (7.64 × 10^3^ copies/mL).

**Figure 1:**
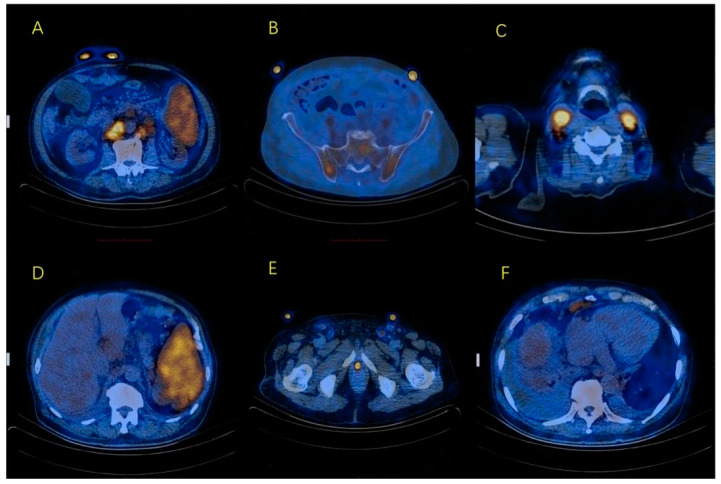
PET/CT showed enlargement of multiple lymph nodes with increased glucose metabolism. (**A**) enlarged retroperitoneal lymph nodes; (**B**) slightly increased glucose metabolism of the pelvis with no bone destruction; (**C**) enlarged cervical lymph nodes; (**D**) splenomegaly with increased glucose metabolism; (**E**) enlargement of bilateral inguinal lymph nodes; (**F**) enlarged lymph nodes in cardiophrenic angle.

After the treatment of IVIG (20 g/d for five days), his EBV-DNA copies decreased (1.48 × 10^5^ copies/mL and 2.54 × 10^3^ copies/mL for serum and peripheral lymphocytes, respectively). However, a progressive decrease in platelets (89 × 10^9^/L) and the enlargement of lymph nodes were observed after three months. His total protein increased to 90.2 g/L with his albumin slightly decreased to 33.3 g/L. His serum immunoglobulin and light chain were also increased (IgG 53.8 g/L(7.2–16.8 g/L), IgA 15.5 g/L(0.7–3.8 g/L), IgM 0.79 g/L(0.6–2.7 g/L), κ 5320 mg/dl(598–1329 mg/dl), λ 2840 mg/dl(280–665 mg/dl)). His thrombin time was prolonged to 20.8 s (12.5–16.5 s). A PET/CT scan showed multiple lymph node enlargements with increased glucose metabolism (Figure 1). The involvement of the tonsils, bone marrow and testicles could not be excluded. His blood immunofixation electrophoresis (IFE) demonstrated polyclonal hypergammaglobulinemia. The urine IFE showed one suspected monoclonal band of the heavy chain of IgG. The reactive plasma cells accounted for 39% when a re-examination of the bone marrow smear of most of them showed mature morphology. The ultrasound showed bilateral PE, which was more severe on the right side. Then the ultrasound-guided thoracic catheterization was performed on the right side. The PE was sent for examination, which revealed its nature as between transudate and exudate.

**Figure 2:**
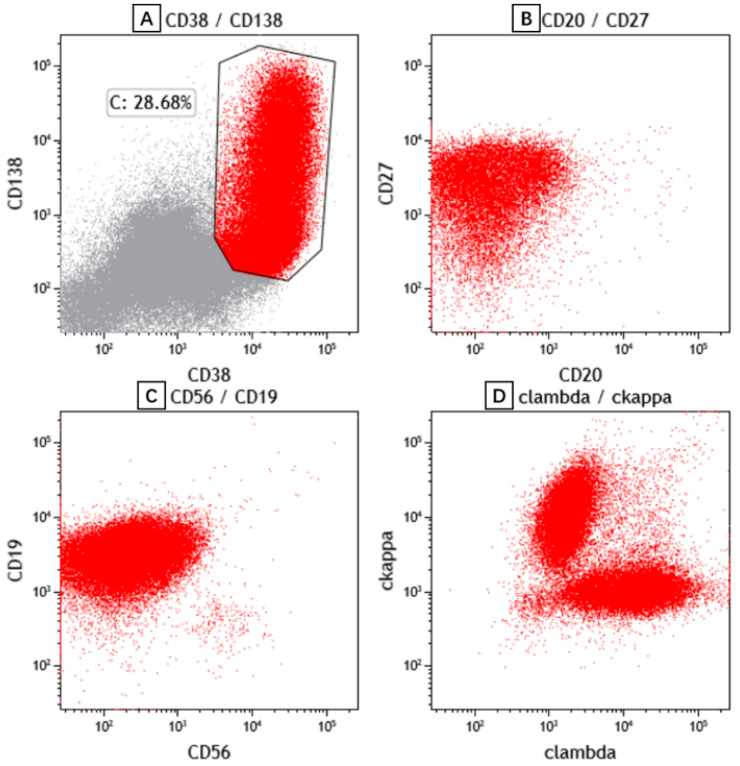
Representative scatter plots of bone marrow aspiration flow cytometric immunophenotyping. The plasma cells express CD38, CD138 (**A**), CD27 (**B**), CD19 (**C**) and polyclonal immunoglobulin light chain kappa and lambda (**D**).

**Figure 3:**
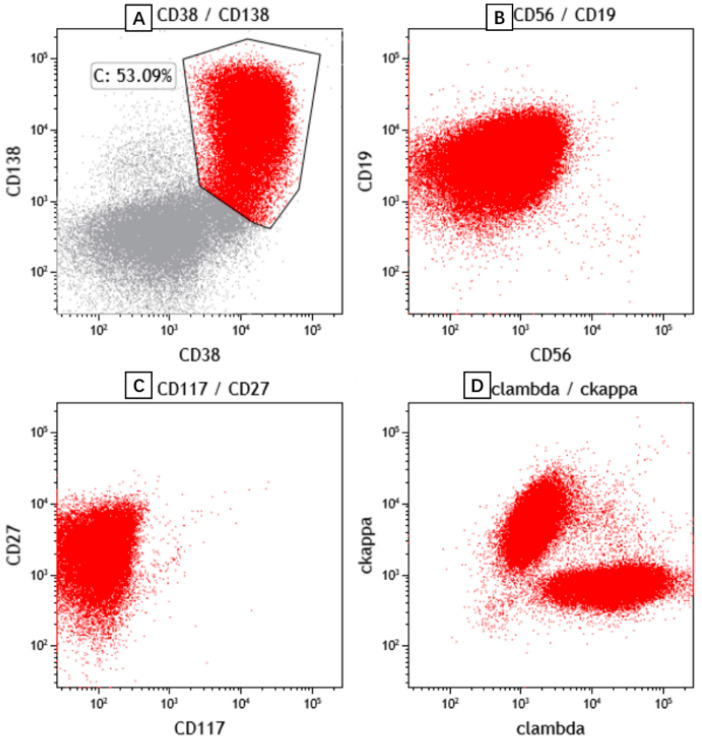
Representative scatter plots of pleural effusion flow cytometric immunophenotyping. The plasma cells express CD38, CD138 (**A**), CD19 (**B**), CD27 (**C**) and polyclonal immunoglobulin light chain kappa and lambda (**D**).

**Figure 4:**
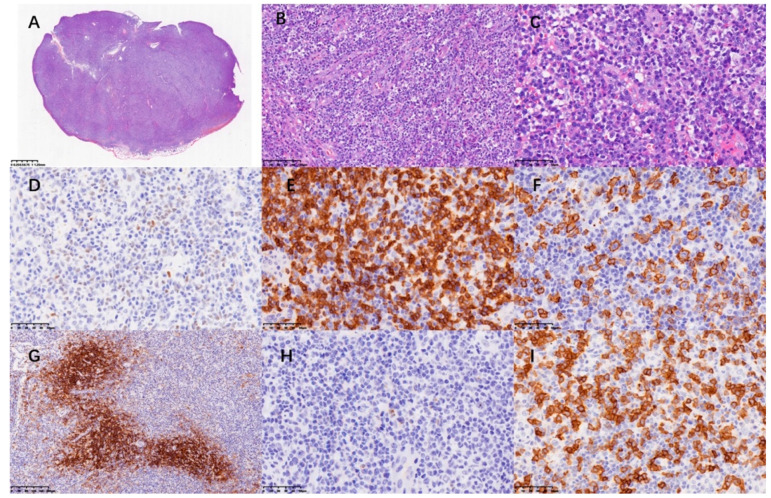
(**A**–**C**) Biopsy of left cervical lymph node showed effacement of architecture and diffuse hyperplasia of medium-large-sized heterogenous lymphocytes with oval nuclei and clear or faintly eosinophilic cytoplasm. The hyperplasia of HEV and plasma cells and infiltrates of eosinophils were also observed. The neoplastic cells were positive for Bcl-6 (**D**), CD3 (**E**) and PD-1 (**I**) in IHC. CD20 (**F**) stood for B cells and CD21 (**G**) labeled follicular dendritic cell meshwork. CXCL13 (**H**) was negative.

The patient’s peripheral blood, bone marrow aspiration (Figure 2) and PE (Figure 3) were subjected to morphological and flow cytometry analyses. The cytogram showed that the populations of plasma cells were 21.1%, 35.2% and 44% respectively. Flow cytometry showed these plasma cells had no abnormal phenotype or restricted light chain expression. A re-examination of the patient’s cervical lymph node showed the effacement of architecture and diffuse hyperplasia of medium-to-large-sized heterogenous lymphocytes with oval nuclei and clear or faintly eosinophilic cytoplasm. The hyperplasia of HEVs and plasma cells and infiltrates of eosinophils were also observed. The atypical medium-to-large cells were positive for CD3 and CD4. Some of these cells also exhibited BCL-6+ and PD-1+, indicating the phenotype of follicular helper T cell (TFH). Some scattered large immunoblasts were CD20-positive. The EBER in situ hybridization test was negative. CD21-positive cells were mainly located in the follicular dendritic cell meshwork. CD56, CXCL13 and CD10 were negative. Ki67 was 70% (Figure 4). The PCR+capillary electrophoresis revealed a positive for the rearrangement of IgH, TCRβ and TCRγ.

Finally, he was diagnosed with AITL and was treated with VP16, dexamethasone and lenalidomide. Unfortunately, this patient died of septic shock and respiratory failure caused by pneumonia two months after diagnosis.

## 3. Discussion and Conclusions

In this case, we present a patient with AITL with exuberant polyclonal plasmacytosis not only in his peripheral blood and bone marrow but also in his PE. Although reactive plasma cells in peripheral blood and/or bone marrow are rare conditions in AITL patients and have been reported before [7,8,11], plasmacytic PE has never been observed before this case. Before the results of the flow cytometry, this presentation offered us a conundrum of plasma cell leukemia. However, the polyclonality of PC revealed by the flow cytometry rules out our initial opinion.

Although reactive plasmacytosis in peripheral blood seems to be a rare condition, it contains a variety of etiologies, including infections, autoimmune diseases and malignancies [12]. Peripheral polyclonal plasmacytosis has been observed in many kinds of infections, such as the hepatitis A virus, Epstein–Barr virus and Parvovirus B19 [12,13,14]. Reactive plasmacytosis in bacterial infections has also been observed [15,16]. This phenomenon has also been observed in autoimmune diseases such as Kawasaki disease and primary Sjogren’s syndrome [17,18]. While in lymphomas, circulating exuberant polyclonal plasmacytosis has only been observed in AITL and several cases have been reported [7,8,19]. Although the patient had a history of EBV infection, ISH showed negative EBER. A similar case was reported before [20]. It may indicate the EBV negativity of this lymph node, or just the false-negative result because of the ISH procedure. The relationship between AITL and reactive plasmacytosis is not clear yet. It is reckoned that it is associated with cytokines, such as IL-6 and IL-10 [7]. Kelsey Sokol et al. believed that the history of EBV infection would also contribute to this condition [19]. Further studies need to be performed to reasonably explain this phenomenon.

Pleural effusions are common complications of non-Hodgkin lymphomas (NHLs) with up 20% prevalence [21]. It was reported that 41.5% of patients with AITL had malignant pleural effusion [22]. Plasmacytic pleural effusions are rare conditions. Research by Ja min Byun et al. showed that among 80 patients with multiple myeloma who had pleural effusion, only 7 of them had a pleural effusion that was myelomatous [23]. Before our presentation, reactive plasmacytosis in pleural effusions had only been observed in tuberculosis, marginal B-cell lymphoma and anaplastic plasmacytoma [9,24,25,26]. No plasmacytic pleural effusions have been observed in AITL patients before. In our case, the flow cytometry (FC) showed the marked plasmacytosis in the patient’s peripheral blood; bone marrow aspiration and PE were all polyclonal, which ruled out our diagnosis of plasma cell leukemia. FC is an efficient tool for identifying malignant cells in body effusions [27]. The sensitivity and specificity of FC were both above 80% in distinguishing malignant body fluids [28]. The exuberant reactive plasmacytosis in this case re-emphasized FC as a valuable tool for the differential diagnosis of PE.

The manifestations that can be observed in AITL include generalized lymphadenopathy, hepatomegaly, splenomegaly, systemic B symptoms, rash, polyarthritis, effusions and symptoms related to anemia [3,5,6]. The symptoms in the present case together with increased serum IgG may also resemble human herpesvirus 8 (HHV 8)-related conditions such as Kaposi sarcoma [29,30]. However, the serum HIV test was negative and the HHV-8 staining for the lymph node biopsy was negative. There was no evident support for HHV-8 infection in the present case.

Clinical features such as fever, rash and eosinophilia may be early signs of AITL [31]. However, these features are not characteristic. Research by Hisao Nagashi et al. recruited 15 AITL patients, and 3 of them had marked reactive plasmacytosis in their peripheral blood. Besides the fact that patients with peripheral plasmacytosis had worse performance status, they found no differences in clinical presentations between the two groups of patients [11]. In recently reported cases, AITL patients with plasmacytosis, monoclonal or not, revealed a bad prognosis [19,32]. In addition to our presented case, we reckon that plasmacytosis may indicate a poor prognosis.

The diagnosis of AITL is a challenge that needs combined evidence including clinical features, morphology, immunophenotype and molecular findings. The immunohistochemistry of the present case was positive for Bcl-6 and PD-1, which indicate the presence of follicular helper T cells (TFHs). We did not check ICOS or mutations such RhoA or IDH2, which may be a limitation of our case. Especially, a mutation of RhoA could help in the early diagnosis of AITL [33]. However, based on the typical morphologic features and two of five TFH features, a diagnosis of AITL can be made. [5]

AITL is generally an aggressive malignancy. In a cohort of 282 AITL patients, the five-year overall survival and progression-free survival were 44% and 32%, respectively [34]. There are no sufficient strategies for the management of this disease. Therapies targeting CD20 or PD-1 showed a lack of effectiveness [35,36]. New treatments such as hypomethylating agents and dasatinib showed promising results in recent studies. However, further clinical trials are needed to validate these findings [37,38].

PEs are not uncommon in AITL patients. Research by Park BB et al. reported 41.5% of patients with AITL had malignant pleural effusion [22]. However, few focused on the cytologic components of PEs in AITL. Cases of lymphocytic and eosinophilic PEs have been reported before [39,40]. To our knowledge, plasmacytic PEs have never been observed before. Due to the rarity of this phenomenon, the mechanisms of plasmacytosis in PEs have never been discussed before. It is probably associated with the increased cytokines in Pes. T Mori et al. observed increased IL-6 in the PE of a patient with idiopathic plasmacytic lymphadenopathy and polyclonal hyperimmunoglobulinemia, while his serum IL-6 was normal [41]. Further research is needed to confirm this theory.

We report this clinical case study to expand the spectrum of clinical manifestation of AITL since we are the ones who first observed the exuberant reactive plasmacytosis in the AITL patient’s pleural effusion. We also want to emphasize the significance of FC in the differential diagnosis of body fluids, since without the clonality test, this amount of plasmacytosis will mislead us to the diagnosis of plasma cell leukemia. Besides FC, we did not run other tests to explain the reason for so large a number of reactive plasma cells in PE, and due to the rarity of this phenomenon, the mechanisms remained unknown.

## Data Availability

The data presented in this study are available on request from the corresponding author.
